# Tracing Genetic Images Formed During Evolution

**DOI:** 10.3390/ijms27093864

**Published:** 2026-04-27

**Authors:** Andrzej Kasperski

**Affiliations:** Laboratory of Bioinformatics and Control of Bioprocesses, Institute of Biological Sciences, Department of Biotechnology, University of Zielona Góra, ul. Szafrana 1, 65-516 Zielona Góra, Poland; a.kasperski@wnb.uz.zgora.pl

**Keywords:** artificial neural network, evolution, genetic images, layered model of the evolution of cellular functionalities, organism development, transition, transversion, unified cell bioenergetics

## Abstract

This work introduces an approach to evolutionary analysis in which information encoded in amino-acid sequences is converted into a specific type of image, termed a genetic image. Genetic images derived from the amino-acid sequences of cytochrome b and cytochrome c oxidase subunit I are shown to be suitable for identifying evolutionary similarities between organisms. Furthermore, artificial neural networks are demonstrated to recognize these genetic images, enabling identification of species evolution. The results indicate the similarity of the genetic images of organisms belonging to species that emerged earlier during Earth’s evolutionary history to the genetic images of organisms belonging to species that emerged later. This finding indicates that genetic images are inherited and undergo gradual modification during evolutionary processes. The phenomenon of inheritance and modification of genetic images suggests that evolution tends to change the already existing functionalities of organisms, which allows for the ordering of organisms belonging to different species from ancient forms, through species that appeared successively during evolution, to those belonging to species that have developed more recently, up to *Homo sapiens*. Moreover, unlike analyses based on phylogenetic trees, the method presented in this article does not require computing hypothetical taxonomic units to study evolution. Combined with analyses of the inheritance of genetic images, it can support the interpretations of phylogenetic trees and evolutionary research.

## 1. Introduction

In accordance with the latest works on evolution, new functionalities that are created during organism evolution extend and prevent already existing functionalities [1,2]. Functionalities consist of a set of functions encoded by genetic information that emerge mainly during macroevolution and are subsequently refined during microevolution [1,2]. In accordance with the layered model of the evolution of cellular functionalities, functionalities are located in three layers, i.e., bioenergetic, unicellular, and multicellular layers [2]. The layer of cell bioenergetic functionalities includes the bioenergetic functionalities needed for life that were formed as first functionalities during the evolution of organisms [2]. The activity of the bioenergetic layer functionalities has to be controlled in order to meet cell bioenergetic needs. For this reason, according to the layered model of evolution of cellular functionalities, during the evolution of unicellular organisms, functionalities of the unicellular layer were formed that used, controlled, and extended the cell bioenergetic layer functionalities [2]. During the evolution of multicellular organisms, functionalities of the multicellular layer were formed that could use, control, and extend both the unicellular layer functionalities and the cell bioenergetic layer functionalities [2]. The layer of multicellular functionalities provides complex life functionalities to multicellular organisms [2]. This process of creating, tuning, and assigning functionalities to the appropriate layers can be schematically illustrated in many ways, for example, in the form of the spiral of life functions [1]. Stepwise formation of functionalities during the evolution of organisms can indicate, among others, that in the genetic information of *Homo sapiens* should be visible some functionalities of species that emerged earlier (from common ancestors) during evolutionary history. As a result, the human genome should contain remnants of the evolutionary history that led to the emergence of such a complex organism as humans. Evidence supporting this concept is, for example, a phenomenon occurring during cancer progression, during which uncontrolled activation of ancient functionalities typical for unicellular organisms occurs in cells of multicellular organisms [2,3,4,5,6]. This point of view is also in line with the atavistic theory of cancer [7,8,9,10,11,12,13] (especially, note Figure 1 in [8]).

The amino-acid sequence of proteins is determined by genetic information encoded in the nucleotide sequence of deoxyribonucleic acid (DNA) and, in some cases, ribonucleic acid (RNA). The universal genetic code encodes 20 amino-acids called standard or canonical amino-acids. These standard amino-acids are encoded directly by the codons of the universal genetic code. Amino-acids have a unique name and single- and three-letter abbreviations. It should be added that there are two nonstandard amino-acids encoded by codons that usually function as stop codons, i.e., selenocysteine (U, Sec, encoded by UGA) and pyrrolysine (O, Pyl, encoded by UAG) [14]. However, the incorporation of these nonstandard amino-acids into proteins is rare and occurs by unique synthetic mechanisms [15,16,17,18,19]. For this reason, it can generally be said that using just 20 standard amino-acids, evolution has painted genetic images that constitute unique patterns specific to different species. In this light, the bodies of organisms belonging to different species are encoded by different genetic information, which can be represented as different genetic images.

Genomes of living organisms are enormous, making it difficult to effectively process such vast amounts of information, even using the fastest computers. For this reason, it is important to select a part of the genome that can act as a genetic identifier of the species evolution. As presented by Hebert et al., species can be identified using “DNA barcodes” [20]. The mitochondrial genome of animals is widely considered a more suitable target for analysis than the nuclear genome due to its lack of introns, limited exposure to recombination, and haploid mode of inheritance [20,21]. For species identification and differentiation analyses, various mitochondrial loci can be used, such as 12S rRNA, 16S rRNA, and COII. However, cytochrome b and cytochrome c oxidase subunit I (COI) belong to the most widely used markers. This is due to the fact that cytochrome b and COI exhibit relatively low intraspecific variability (cytochrome b intraspecific variation is similar to that of COI) but show sufficient interspecific variation to enable the estimation of degrees of species relatedness [22]. As demonstrated in numerous published articles, sequences of cytochrome b and COI can represent species and can be used as barcoding tools [20,23]. Cytochrome b was until recently the main locus on the mitochondrial genome used in species discrimination, determining phylogenetic relationships between species and aid in the genus assignment of newly described organisms, but it should be added that the use of COI has recently increased primarily after its adoption by the Barcode for Life Consortium [22,24,25,26,27,28,29,30]. It was, for example, established that the mitochondrial gene cytochrome c oxidase subunit I (COI) can be used for identification of animals and stated that after full development, an identification system based on COI can provide a reliable solution to the species identification problem [20]. The use of COI also has its limitations. Because changes in the COI amino-acid sequence occur more slowly than in any other mitochondrial gene [20,31], COI is highly efficient for discriminating vertebrate and invertebrate species [32,33,34], but it is not suited for discriminating plants and some fungal species [20,35,36,37], because the sequence of the plant COI gene also does not change much. In this work, it is demonstrated that information stored in amino-acid sequences can be analyzed by treating it as an image and that images of the genetic representatives (i.e., images “formed” by cytochrome b and COI amino-acid sequences) can be used to study evolution. These images “formed” by amino-acid sequences are called, in this article, genetic images. The evolution of species occurs on two scales: microevolution and macroevolution [38,39,40,41,42]. Microevolution introduces small changes in the genome over time, enabling species to better adapt to their environment. Macroevolution, in contrast, involves large-scale evolutionary changes that occur as a result of the accumulation of small changes introduced during microevolution [38,43,44,45,46]. In the course of evolution, species undergo changes as a result of successive processes of microevolution and macroevolution, during which old functionalities are stored and prevented and new functions are created and then modified [1,38,39,41]. This perspective additionally supports the idea that inspired and motivated the present study: in genetic images of the genetic representatives of newly emerged species, inherited genetic images of the genetic representatives of species that emerged earlier during Earth’s evolutionary history should be visible.

Nowadays, artificial intelligence-based methods are one of the most important image classification techniques that can be used in real-world scenarios [47]. Applications of an artificial neural network (ANN), as one of the artificial intelligence methods, include, for example, medical image analysis and interpretation (among others, automated electrocardiographic interpretation), electronic signal analysis, drug development, clinical diagnostics and predictions (for example, prediction of cancer progression), and recognition of genome attractors and timestamps [44,48,49,50,51,52,53,54,55]. In accordance with the current research interest, organisms can be considered as attractors in phase space [56]. The term ‘attractor’ refers to a configuration towards which the system evolves over time. Once an attractor is reached, the system remains sufficiently stable to return to this state after perturbations dissipate [57,58,59]. Attractors that trap genomes of organisms are termed ‘genome attractors’ (for additional information, see [44,50]), as they define and stabilize organisms [44,50]. From this perspective, evolution can be viewed as a discontinuous process during which organisms belonging to different species evolve in distinct genome attractors [44,50,60]. That implies that different genetic images have been formed during the evolution of different species and that organisms can be characterized by specific, species-dependent genetic images. In this study, artificial neural networks (ANNs) are applied to recognize genetic images formed during evolution. In particular, amino-acid sequences of cytochrome b and COI have been used to investigate the evolution of selected organisms belonging to different species (see Table A1 in Appendix A.1) by identifying genetic images derived from these sequences.

This article examines the evolution of organisms belonging to different species from a wide evolutionary range, i.e., from unicellular, structurally simple forms to multicellular complex species that emerged relatively recently during Earth’s evolutionary history (see Table A1 in Appendix A.1). In the light of unified cell bioenergetics (UCB), reactive oxygen species (ROS) can be considered one of the factors driving microevolutionary changes at the molecular level, which can optionally lead to macroevolutionary changes [2,44,50,61,62,63]. The life of organisms is driven and characterized, among others, by the occurrence of very complex bioenergetic phenomena. Unified cell bioenergetics (UCB) integrates fundamental bioenergetic phenomena that can occur in eukaryotic cells. In accordance with UCB, mitochondria are charging with NADH during the Krebs cycle, and mitochondria are discharging from NADH in the electron transport chain [2,44,50,61,62,63]. Because the inner mitochondrial membrane is impermeable to NADH, factors affecting the charging of mitochondria and discharging (for example, environmental conditions) can affect the level of mitochondrial NADH [63]. Disturbances in the NADH level, especially the accumulation of NADH, can directly influence organism life, as an increase in the NADH level causes an exponential increase in ROS, resulting in an increase in the probability of random genetic mutations [50,64,65,66]. According to existing theories, the factors that drive the evolution of organisms include random genetic mutations and natural selection [67,68]. It is known that a moderate ROS level has a positive effect on cells, affecting, among others, a number of cellular processes through transcriptional regulation [61,69]. In normal cells, the ROS level is moderate, which means that ROS stimulates the life processes of these cells [44,50]. According to UCB, disturbances (perturbations) in cell bioenergetics causing disturbances in the amount of NADH and, as a result, disturbances in the rate of ROS generation can be considered one of the factors stimulating the evolution of normal organisms [50,61,63]. These disturbances in cell bioenergetics can be caused, for example, by changes in the environment and can result in small ROS-induced changes in DNA, thus driving microevolution. However, the accumulation of small ROS-induced changes in DNA over a long period of time of microevolution can also lead to macroevolution. It should be noted that disturbances (perturbations) in cell bioenergetics may also elevate a ROS level to high values, resulting in an increased risk of cancer transformation and then cancer progression [61,70,71]. In this light, phenomena occurring during the evolution of normal organisms as well as transformed cells (including macroevolution and microevolution) can be studied using unified cell bioenergetics, and in view of UCB, disturbances (perturbations) of cell bioenergetics can be considered as the main factor driving evolution [50]. These bioenergetic disturbances and the resulting perturbations in ROS level can contribute to evolutionary changes at the molecular level by inducing nucleotide substitutions in amino-acid-encoding codons. Such substitutions occur during evolution and can be of the transition type (i.e., purine–purine or pyrimidine–pyrimidine interchanges) or the transversion type (i.e., purine–pyrimidine interchanges) [72]. A common pattern in molecular evolution is that nucleotide transitions are favored several times over transversions [73]. When this pattern occurs among amino-acid replacements, one of the possible explanations is that, in the light of natural selection, the transitions have a more conservative effect on proteins [73]. The other exemplary explanations for why natural selection favors amino-acid replacements via transitions can be that (a) transitions are less severe considering the chemical properties of the original and mutant amino-acids, (b) transitions tend to cause changes that preserve the chemical properties of amino-acids and (c) transversions cause the biochemical difference in the protein product to tend to be greater compared to the impact of transitions on proteins [73,74,75,76]. Therefore, determining the number of transitions and transversions (especially those causing amino-acid replacements) in the examined sequences can be used to analyze evolutionary relationships among the studied organisms, as demonstrated in this work, providing a method that supports the recognition of genetic images. Additionally, in this study, the interpretation of the results is further examined using phylogenetic trees and rRNA.

## 2. Results and Discussion

During evolution, species undergo changes that lead to improved adaptation to their environment. At the outset of this study, it was hypothesized that genetic images “formed” by the genetic representatives (i.e., “formed” by cytochrome b and COI amino-acid sequences) of species that emerged later in Earth’s evolutionary history would contain traces of inherited genetic images from species that appeared earlier (see Section 3.2). This inheritance of genetic images was examined both when the recognition process was initiated with a relatively recently evolved species (i.e., *Homo sapiens*) and when it was initiated with ancient organisms (i.e., bacteria).

The concatenation of amino-acid sequences of cytochromes was performed to generate larger images, thereby increasing the sensitivity of the recognition process. Additionally, to enable comparison of results, images created solely by cytochrome b and solely by COI were analyzed.

### 2.1. Recognition of Genetic Images When the Uncovering Process Starts from the Top of Evolution

Starting the analysis with Human (*Homo sapiens*), who emerged relatively recently during Earth’s evolutionary history [77], the Human genetic image (i.e., the image “formed” by the genetic representatives of *Homo sapiens*) is expected to show similarities to the genetic images of species that emerged earlier during evolution. As expected, the most visible similarity of the Human genetic image should be to the genetic images of organisms belonging to species evolutionarily closest to Human. It should also be possible to recognize similarities between the Human genetic image and the genetic images of organisms belonging to species that are evolutionarily more distant from Human, although this visibility is expected to gradually decrease toward taxa that diverged earlier in evolutionary history. This implies that the similarity recognized by the ANNs can be interpreted as a measure of the inheritance of genetic images.

#### 2.1.1. Recognizing the Similarity of the Human (*Homo sapiens*) Genetic Image to the Genetic Images of Organisms Belonging to Different Species

The idea presented in this article has been demonstrated using 32 organisms belonging to different species, including Human (*Homo sapiens*) (see Table A1 in Appendix A.1). To recognize the similarity of the Human (*Homo sapiens*) genetic image to the genetic images of other organisms, sequences of all organisms from the initial set of organisms (see Table A1 in Appendix A.1) except Human (*Homo sapiens*) were used in the ANN teaching process:

(a) Five artificial neural networks (ANNs) were taught using the concatenated cytochrome b and COI amino-acid sequences, and then the Human (*Homo sapiens*) concatenated cytochrome b and COI amino-acid sequences were recognized five times by these ANNs, followed by the calculation of average values of recognition;
(b) Five ANNs were taught using the cytochrome b amino-acid sequences, and then the Human (*Homo sapiens*) cytochrome b amino-acid sequence was recognized five times by these ANNs, followed by the calculation of average values of recognition;
(c) Five ANNs were taught using the COI amino-acid sequences, and then the Human (*Homo sapiens*) COI amino-acid sequence was recognized five times by these ANNs, followed by the calculation of average values of recognition.

In the first step of the process, the similarity of the Human (*Homo sapiens*) genetic image to the Chimpanzee genetic image and the Gorilla genetic image was clearly recognized (Table 1). To ensure the readability of the results presentation, this work depicts average values of recognition that are greater than or equal to the recognition threshold set to 0.01 (additionally, see Section 3.2).

**Remark** **1.** *In the column “Organisms whose genetic images were recognized”, the organisms whose genetic images have been recognized by ANNs are presented. In the column “cytochrome b and COI”, results (i.e., average values of recognition by ANNs and the number of “R”, “#”, “$”, and “-” positions (see Section 3.3)) for concatenated cytochrome b and COI amino-acid sequences are presented. In the column “cytochrome b”, results for cytochrome b amino-acid sequences are presented. In the column “COI”, results for COI amino-acid sequences are presented. The number of “R”, “#”, “$”, and “-” positions was calculated by comparing Human (Homo sapiens) amino-acid sequences (i.e., concatenated cytochrome b and COI amino-acid sequences, cytochrome b amino-acid sequences, and COI amino-acid sequences) with amino-acid sequences of the organisms whose genetic images have been recognized by ANNs (i.e., with the concatenated cytochrome b and COI amino-acid sequences, cytochrome b amino-acid sequence, and COI amino-acid sequence of the organisms whose genetic images have been recognized by ANNs).*

As it is visible in Table 1, the Chimpanzee genetic image is much better visible (with average values of recognition equal to 0.4833, 0.3865, and 0.8655 recognized accordingly by ANNs taught using concatenated cytochrome b and COI (the first value), cytochrome b (the second value), and COI (the third value) amino-acid sequences) than the Gorilla genetic image (0.1412, 0.1201, and 0.0875). From this point of view, it can be said that the Gorilla genetic image is visible in the background of the Chimpanzee genetic image.

**Remark** **2.** *The Chimpanzee genetic image is an example of a foreground genetic image. The Gorilla genetic image is an example of a background genetic image.*

It should be noted that the differences in the numbers of “R” positions for Chimpanzee and Gorilla are relatively small (accordingly, 862 and 854; 355 and 350; 507 and 504, see Table 1), which indicates that ANNs are able to more clearly recognize differences between these two organisms than the number of homologous comparisons suggests. It is also clearly visible that the numbers of “#” positions are bigger than the numbers of “$” positions (for Chimpanzee, accordingly, 24 and 3; 20 and 2; 4 and 1, for Gorilla, 26 and 8; 19 and 7; 7 and 1), and the numbers of “-” positions are small, which indicates a relatively close evolutionary relationship between these two organisms and Human.

Because the ANNs recognized only two genetic images when recognizing the Human genetic image, it was likely that these recognized images obscured the genetic images of other organisms used to teach the ANNs (see Remark 3). To uncover the invisible genetic images formed during evolution, the two organisms whose genetic images had been recognized were additionally removed from the teaching set of organisms. After this removal, the ANNs were taught once again, and the Human genetic image was recognized by the newly taught ANNs. The results of this recognition are presented in Table 2.

**Remark** **3.** *In order to recognize the genetic images that are invisible, it is necessary to remove those that obscure them. From this perspective, such invisible images must be “uncovered”. For this reason, the process and method presented in this work are referred to as the uncovering process and the uncovering method, respectively.*

In this step of uncovering genetic images, the genetic image of only one organism was recognized (i.e., African bush elephant, see Table 2). This result can be confirmed by generated phylogenetic trees (see Section 2.4), where Chimpanzee, Gorilla and African bush elephant are presented close to Human. It should be noted that the average values of recognition of the African bush elephant genetic images are small (i.e., 0.0148, 0.0114, unrecognized), where “unrecognized” means that it was impossible to recognize any genetic image (i.e., the average value recognized by ANNs taught using COI amino-acid sequences was less than threshold 0.01), which indicates a big evolutionary distance between Human and African bush elephant. It should also be noted that, compared to Chimpanzee and Gorilla, the number of homologous positions (i.e., “R” positions) dropped noticeably and the number of positions with two or three point mutations in the codons of the compared amino-acids (i.e., “-” positions) increased noticeably, which indicates an increase in the evolutionary distance between Human and African bush elephant compared to evolutionary distances between Human and Chimpanzee and Human and Gorilla.

Because the ANNs recognized only one genetic image when recognizing the Human genetic image, it was likely that this one recognized genetic image obscured the genetic images of other organisms used to teach the ANNs. As it was made in the previous step, in order to uncover other genetic images formed during evolution, this one organism whose genetic image had been recognized (i.e., African bush elephant) was additionally removed from the teaching set of organisms. After this removal, the ANNs were taught once again, and the Human genetic image was recognized by the newly taught ANNs. The results of the recognition are presented in Table 3.

In this step of uncovering genetic images, the genetic image of only one organism was recognized (i.e., Gray wolf, see Table 3). That means that the ANNs taught using only cytochrome b amino-acid sequences were unable to recognize any similarities in this step. It should be noted that the ANNs taught using only COI amino-acid sequences were unable to recognize any similarities in the previous step. For this reason, in this step, the results are presented for the ANNs taught using concatenated cytochrome b and COI amino-acid sequences and the ANNs taught using only cytochrome b amino-acid sequences.

**Remark** **4.** *Because of the longest maintenance of the ability to recognize genetic images, the system that consists of ANNs taught using concatenated cytochrome b and COI amino-acid sequences was named the main recognition system, and results obtained using this system are considered leading results. In the case of recognizing more than one genetic image by the main recognition system, the first recognized image is called a foreground genetic image, and the other recognized images are called background genetic images (additionally, see Remark 2).*

It should be noted that, similarly to the previous step, the average value of recognition of the Gray wolf genetic image was small (i.e., 0.0109, see Table 3), and comparing to Chimpanzee and Gorilla, the number of homologous positions (i.e., “R” positions) dropped noticeably and the number of positions with two or three point mutations in the codons of the compared amino-acids (i.e., “-” positions) increased noticeably.

Because the main recognition system (see Remark 4) recognized only one genetic image when recognizing the Human genetic image, it was likely that this one recognized genetic image obscured the genetic images of other organisms used to teach the ANNs. As it was made in the previous steps, in order to uncover other genetic images formed during evolution, this one organism whose genetic image had been recognized (i.e., Gray wolf) was additionally removed from the teaching set of organisms. After this removal, the ANNs were taught once again, and the Human genetic image was recognized by the newly taught ANNs. The results of the recognition are presented in Table 4.

In step 4A of uncovering genetic images, the main recognition system (see Remark 4) retained its ability to recognize genetic images, and once again, the genetic image of only one organism was recognized by this system (i.e., Chinese hare, see Table 4). Since it was likely that the Chinese hare genetic image obscured genetic images of other organisms used to teach the ANNs, this one organism whose genetic image had been recognized (i.e., Chinese hare) was additionally removed from the teaching set of organisms in order to uncover other genetic images formed during evolution. After this removal, the ANNs were taught once again, and the Human genetic image was recognized by the newly taught ANNs. In this step, the main recognition system also lost the ability to recognize genetic images. In order to continue the process of uncovering genetic images formed during evolution, it became necessary to change the organism whose genetic image would be recognized.

#### 2.1.2. Recognizing the Similarity of the Chimpanzee (*Pan troglodytes*) Genetic Image to the Genetic Images of Organisms Belonging to Different Species

Because it was impossible to recognize the Human (*Homo sapiens*) genetic image using the main recognition system, the next steps of the process were continued for the evolutionary closest organism to Human (*Homo sapiens*) that was recognized by the main recognition system (see Remark 4), i.e., Chimpanzee (*Pan troglodytes*) (see Table 1). Genetic images of two organisms were recognized after changing the reference organism from Human to Chimpanzee (Table 5).

It is visible that in step 5A, in addition to the foreground genetic image (i.e., the Asian rat genetic image), one background genetic image (i.e., the Brown bear genetic image) was recognized by the main recognition system (Table 5). Asian rat is marked by “*” as the first organism for which a foreground genetic image was recognized after the change of the reference organism from Human to Chimpanzee (Table 5).

Next, the algorithm presented in the previous steps was repeated, and uncovering the genetic images of subsequent organisms gradually occurred. The obtained results are presented in Table A2, Table A3 and Table A4 in Appendix A.1 and shortly discussed. In step 6A of the process, in addition to the foreground genetic image (i.e., the Golden hamster genetic image), two background genetic images were recognized by the main recognition system (Table A2 in Appendix A.1). The Bactrian camel genetic image was recognized as the first background genetic image, and the Horse genetic image was recognized as the second background genetic image (Table A2 in Appendix A.1). In step 7A of the process, the Four-horned antelope genetic image was recognized as the foreground genetic image. Moreover, two background genetic images were recognized, i.e., the Domestic sheep genetic image as the first background genetic image and the Blue whale as the second background genetic image (Table A3 in Appendix A.1). In step 8A of the process, the Maroon-fronted parrot genetic image was recognized as the foreground genetic image. Moreover, two background genetic images were recognized, i.e., the Atif’s Lycian salamander genetic image as the first background genetic image and the Indo-Pacific crocodile as the second background genetic image (Table A4 in Appendix A.1). In order to uncover the next genetic images, the organisms (whose genetic images had been recognized) were additionally removed from the teaching set of organisms in step 9A. After this removal, the ANNs were taught once again, and the Chimpanzee genetic image was recognized by the newly taught ANNs. The results of the recognition are presented in Table 6.

**Figure 1:**
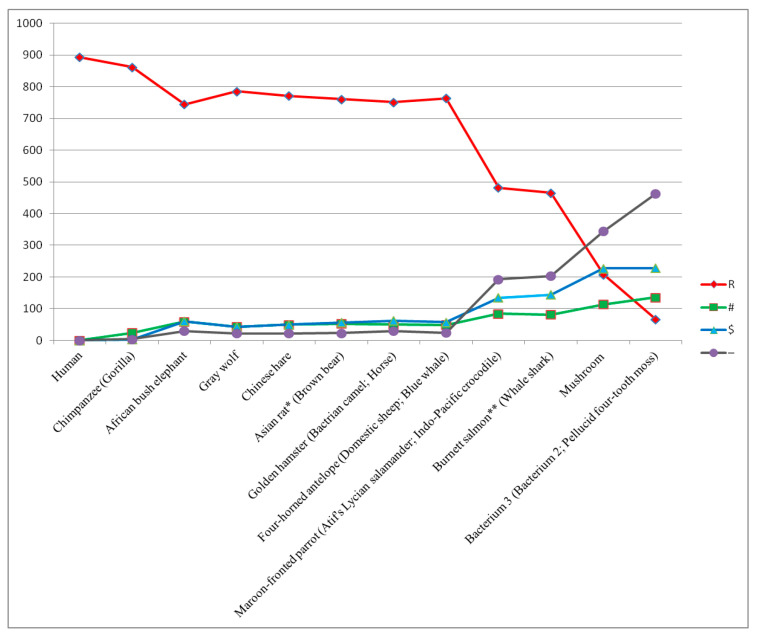
Result of uncovering genetic images when the uncovering process started from the top of evolution. Organisms whose genetic images were recognized are presented on the X axis. Organisms whose background genetic images were recognized are presented in brackets. Asian rat and Burnet salmon as threshold organisms are marked by “*” and “**” accordingly. The number of homologous (“R”), semihomologous (“#”, “$”), and other “-” positions is established when comparing concatenated cytochrome b and COI amino-acid sequences of Human (*Homo sapiens*) with concatenated cytochrome b and COI amino-acid sequences of organisms whose foreground genetic images were recognized.

In step 9A of the process, the Burnett salmon genetic image was recognized as the foreground genetic image and the Whale shark genetic image as the background genetic image (Table 6). In the next two steps of the process, the Mushroom (*Phallus echinovolvatus*) and Bacterium 3 (*Shewanella xiamenensis*) genetic images were recognized as the foreground genetic images, and the Bacterium 2 (*Pseudomonas flexibilis*) and Pellucid four-tooth moss (*Tetraphis pellucida*) genetic images were recognized as the background genetic images (see Table A5 and Table A6 in Appendix A.1). All these uncovered organisms are characterized by a small number of homologous positions (i.e., “R” positions) compared to the number of “R” positions determined for Burnett salmon. For this reason, Burnet salmon (marked by “**”) can be considered the threshold organism after which the number of homologous positions (i.e., “R” positions) suddenly drops (below 25%) and the uncovering process is quickly finished by attaining the most structurally simple organisms from the considered set of organisms (i.e., bacteria) (see Figure 1). In Figure 1, all organisms whose genetic images were recognized by the main recognition system when the uncovering process started from the top of evolution are presented.

### 2.2. Recognition of Genetic Images When the Uncovering Process Starts from the Bottom of Evolution

In the previous part of this work, it was presented that in the genetic images of the genetic representatives of species that emerged later during Earth’s evolutionary history, there are visible inherited genetic images of organisms that emerged earlier during evolutionary history. This part of the study demonstrates that genetic images of organisms belonging to species that appeared earlier in Earth’s evolutionary history can be recognized in the genetic images of organisms belonging to more recently evolved species. To illustrate this phenomenon, the recognition process was initiated with Bacterium 3 (*Shewanella xiamenensis*), as a representative of the ancient organisms, whose recognition of a foreground genetic image stopped the previous process of recognition of genetic images (i.e., when the uncovering process started from the top of evolution).

#### Recognizing the Similarity of the Bacterium 3 (*Shewanella xiamenensis*) Genetic Image to the Genetic Images of Organisms Belonging to Different Species

To recognize the similarity of Bacterium 3 (*Shewanella xiamenensis*) genetic images to the genetic images of organisms belonging to different species, the sequences of all organisms from the initial set of organisms (see Table A1 in Appendix A.1) except Bacterium 3 (*Shewanella xiamenensis*) were used in the ANN teaching process, i.e., five artificial neural networks (ANNs) were taught using the concatenated cytochrome b and COI amino-acid sequences. Then, after teaching, the Bacterium 3 (*Shewanella xiamenensis*) concatenated cytochrome b and COI amino-acid sequences were recognized five times by these ANNs, followed by the calculation of average values of recognition.

The algorithm presented in the previous part of the article was repeated, and uncovering the genetic images of subsequent organisms gradually occurred. The obtained results are presented and briefly discussed. In the first two steps of uncovering genetic images, the highest recognized similarities by the main recognition system (see Remark 4) were similarities to Bacterium 1 (*Nitrobacter vulgaris*) and Bacterium 2 (*Pseudomonas flexibilis*) (Table 7 and Table 8). Although these recognized similarities were expected, they were also very small (i.e., less than 0.01), which can be explained by the high genetic variability of bacteria [78]. Because of the very small recognized similarities, the genetic images of Bacterium 1 and Bacterium 2 are presented as background genetic images of Bacterium 3.

In step 3B, Bacterium 2 (*Pseudomonas flexibilis*) was additionally removed from the teaching set of organisms, and the ANNs were taught again, followed by recognition of the Bacterium 3 genetic image by the newly taught ANNs. The results of the recognition are presented in Table 9.

In the consequent four steps, the foreground genetic images of three plants (i.e., Grass (in step 3B, Table 9), Date palm (step 4B, Table A7 in Appendix A.1), and Pellucid four-tooth moss (step 5B, Table A8 in Appendix A.1), and Chinese scorpion (step 6B, Table A9 in Appendix A.1)) were recognized. Moreover, the background genetic images of Atlantic awning clam and Sponge (step 5B, Table A8 in Appendix A.1) and Fly, Mushroom, and Wasp (step 6B, Table A9 in Appendix A.1) were recognized.

In step 7B, the ANNs were taught again (after removing the organisms whose genetic images had been recognized), and the Bacterium 3 genetic image was recognized by the newly taught ANNs. The results of the recognition are presented in Table 10.

**Figure 2:**
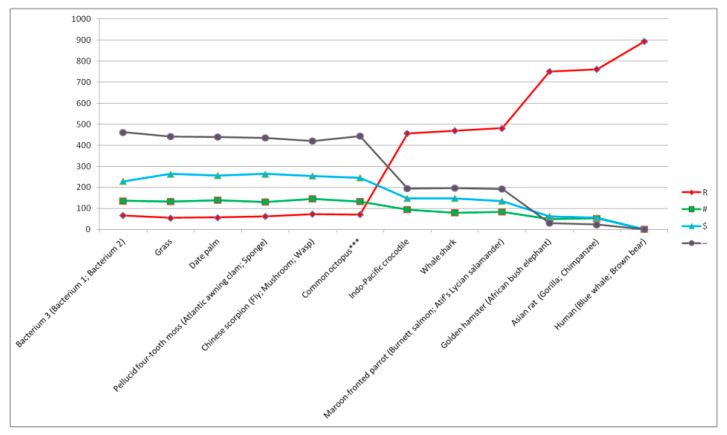
Result of uncovering genetic images when the uncovering process started from the bottom of evolution. Organisms whose genetic images were recognized are presented on the X axis. Organisms whose background genetic images were recognized are presented in brackets. Common octopus as the threshold organism is marked by “***”. The number of homologous (“R”), semihomologous (“#”, “$”), and other “-” positions is established when comparing concatenated cytochrome b and COI amino-acid sequences of Human (*Homo sapiens*) with concatenated cytochrome b and COI amino-acid sequences of organisms whose foreground genetic images were recognized.

In step 7B of uncovering genetic images, the main recognition system (see Remark 4) was able to recognize the genetic image of only one organism, i.e., Common octopus (Table 10). In the next steps (i.e., steps 8B–13B), subsequent organisms were step-by-step recognized, leading to the uncovering of the Human (*Homo sapiens*) genetic image. These recognized organisms are characterized by a high number of homologous positions (i.e., “R” positions) compared to the number of “R” positions determined for Common octopus (see Table A10, Table A11, Table A12, Table A13, Table A14 and Table A15 in Appendix A.1). The uncovering process is finished by recognizing the Human (*Homo sapiens*) genetic image (Table A15 in Appendix A.1). Common octopus (marked by “***”) can be considered the threshold organism because, after uncovering this organism, continuation of the uncovering process leads to a quick increase in the number of homologous positions (i.e., “R” positions) exceeding 50% (see Figure 2), which makes the uncovering process using a very structurally simple organism (i.e., a bacterium) as a reference organism less credible. In Figure 2, all organisms whose genetic images were recognized by the main recognition system when the uncovering process started from the bottom of evolution are presented.

In Figure 2, it is visible that the number of homologous (“R”), semihomologous (“#”, “$”), and other (“-”) positions for all organisms from Bacterium 3 to Common octopus is practically constant, which confirms that Common octopus can be considered the threshold organism.

### 2.3. Reconstruction of the Virtual Line of Inheritance of Genetic Images When the Uncovering Process Starts from the Top and Bottom of Evolution

**Figure 3:**
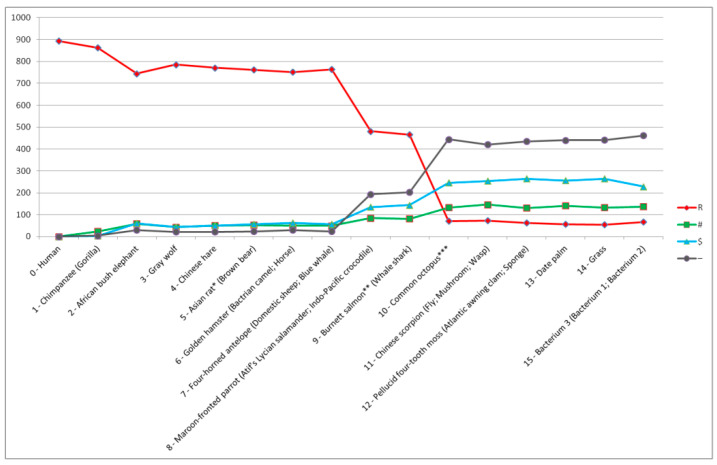
Result of uncovering genetic images presented in the form of a reconstructed virtual line of inheritance of genetic images. Organisms whose genetic images were recognized are presented on the X axis. Human is at position 0, the first organism for which a foreground genetic image was recognized (i.e., Chimpanzee) is at position 1, the second organism for which a foreground genetic image was recognized (i.e., African bush elephant) is at position 2, and so on. Organisms whose background genetic images were recognized are presented in brackets. Asian rat, Burnet salmon, and Common octopus as threshold organisms are marked by “*”, “**”, and “***” accordingly. The number of homologous (“R”), semihomologous (“#”, “$”), and other “-” positions is established when comparing concatenated cytochrome b and COI amino-acid sequences of Human (*Homo sapiens*) with concatenated cytochrome b and COI amino-acid sequences of organisms whose foreground genetic images were recognized.

Figure 3 presents the result of uncovering genetic images in the form of a reconstructed virtual line of inheritance of genetic images. The reconstruction of the virtual line (i.e., the line along which inherited genetic images of organisms are visible) was made possible by starting the uncovering process from the top and from the bottom of evolution, taking into account threshold organisms. Starting from Human (*Homo sapiens*), the genetic images of all organisms to Chinese hare (*Lepus sinensis*) were recognized using the Human genetic image (Figure 3). The genetic images of all organisms from the first threshold organism (i.e., Asian rat (*Rattus tanezumi*)) to the second threshold organism (i.e., Burnett salmon (*Neoceratodus forsteri*)) were recognized using the Chimpanzee (*Pan troglodytes*) genetic image. Starting from Bacterium 3 (*Shewanella xiamenensis*), the genetic images of all organisms to the third threshold organism (i.e., Common octopus (*Octopus vulgaris*)) were recognized using the Bacterium 3 genetic image.

**Remark** **5.** *While determining the virtual line of inheritance of genetic images, the genetic images of all organisms from the initial set of organisms (see Table A1 in Appendix A.1) were recognized. The inheritance of genetic images is visible from bacterium to Human (Homo sapiens), i.e., from a unicellular, structurally simple organism to a very complex multicellular organism along this line. The reconstructed virtual line of inheritance of genetic images (with the grouping of mammals, plants, and bacteria, a visible relationship between fungi and animals, and the placement of birds (with recognized amphibian and reptile background genetic images) between mammals and fish) is broadly consistent with evolutionary relationships reported in the literature and supported by molecular clock analyses and fossil-calibrated divergence times [79,80,81,82,83,84,85,86,87]*.

From Figure 3, it is visible, among others, that very distant organisms are characterized by a larger number of transversions (i.e., “$” positions) compared to the number of transitions (i.e., “#” positions) and a small number of homologous positions (i.e., “R” positions). As the evolutionary distance between organisms decreases, the number of homologous (“R”) positions increases, and the difference between the number of transversions (“$”) and the number of transitions (“#”) decreases. A further decrease in the evolutionary distance causes the number of transitions (“#”) to reach the number of transversions (“$”) and then begins to exceed the number of transversions (“$”).

### 2.4. Confirmation of the Reconstructed Virtual Line of Inheritance of Genetic Images Using Phylogenetic Trees

In general, a phylogenetic tree is a diagrammatic representation of the evolutionary relationships among various taxa [88]. It is a branching diagram composed of nodes and branches (where the nodes represent taxonomic units and the branches represent the time estimate of the evolutionary relationships among the taxonomic units) [88]. In a phylogenetic tree, the terminal nodes (i.e., leaves) represent the operational taxonomic units. The operational taxonomic units are the actual objects being compared (i.e., the protein sequences of 32 organisms in the case presented in this article), whereas the internal nodes represent inferred hypothetical taxonomic units [88].

**Figure 4:**
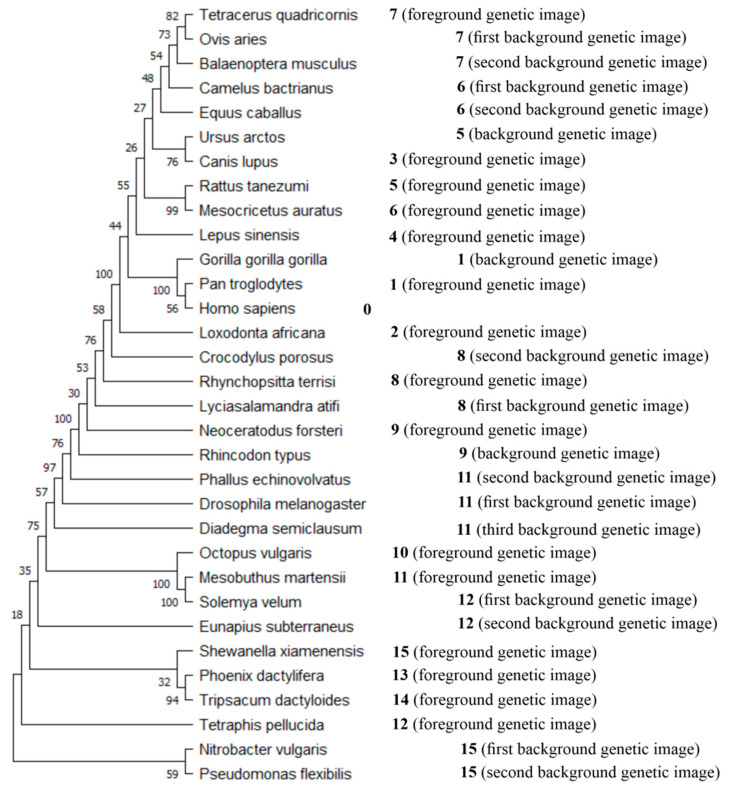
Cladogram generated based on the concatenated cytochrome b and COI amino-acid sequences of 32 organisms. The positions of organisms in the virtual line of inheritance of genetic images (see Figure 3) are presented to the right of the tree leaves (where Human (*Homo sapiens*) is at position 0 and Bacterium 3 (*Shewanella xiamenensis*) is at position 15). Moreover, information about the type of genetic image (i.e., foreground or background genetic image) recognized at each position is presented; for example, at position 8, the genetic image of *Rhynchopsitta terrisi* was recognized as a foreground genetic image, the genetic image of *Lyciasalamandra atifi* was recognized as the first background image, and *Crocodylus porosus* was recognized as the second background genetic image (see Table A4 in Appendix A.1).

To generate a phylogenetic tree, the Maximum Likelihood method (i.e., the ML method) was used, which allows for the generation of rooted trees (additionally, see Section 3.4) [89,90]. The tree presented in Figure 4 was generated based on the concatenated cytochrome b and COI amino-acid sequences of 32 organisms examined in this work (see Table A1 in Appendix A.1). The method presented in this article allows, unlike analyses using phylogenetic trees, to study the evolutionary similarities between operational taxonomic units without calculating hypothetical taxonomic units. The 32 organisms examined in this article, as actual objects being compared, are represented by leaves of the phylogenetic tree (Figure 4). In this light, it was interesting to compare the distribution of leaves in the generated phylogenetic tree with the distribution of organisms along the virtual line of inheritance of genetic images. In order to facilitate the analysis, the positions of organisms in the virtual line of inheritance of genetic images (the numbers to the right of the tree leaves) and information about the recognized type of genetic image (i.e., foreground or background genetic image) for each position are presented in Figure 4.

In Figure 4, it is visible that Chimpanzee (*Pan troglodytes*) and African bush elephant (*Loxodonta africana*) are the first and second organisms for which foreground genetic images were recognized, and these two organisms are located in the tree close to Human. As expected, it is visible in Figure 4 that organisms whose genetic images were recognized as background genetic images are located close to an organism whose genetic image was recognized as a foreground genetic image (for example, considering position 8, the organisms whose genetic images were recognized in step 8A of the process (see Table A4 in Appendix A.1), i.e., *Lyciasalamandra atifi* and *Crocodylus porosus*, are located very close to *Rhynchopsitta terrisi*). Moreover, starting with Human, the organisms whose genetic images were recognized as foreground genetic images are located increasingly further and further upwards (except African bush elephant (*Loxodonta africana*)) from Human (*Homo sapiens*) in the phylogenetic tree (Figure 4). Then, after reaching the last mammals (i.e., Four-horned antelope (*Tetracerus quadricornis*), Domestic sheep (*Ovis aries*), and Blue whale (*Balaenoptera musculus*)) from the considered set of organisms (see Table A1 in Appendix A.1), the organisms whose genetic images were recognized as foreground genetic images are located increasingly further and further downwards from Human (*Homo sapiens*) in the phylogenetic tree (Figure 4). In this light, it is visible that the distribution of leaves (which represent 32 organisms examined in this work) is in line with the distribution of organisms along the virtual line of inheritance of genetic images.

It should be added that the methods of generation of phylogenetic trees (the Neighbor Joining (NJ), Maximum Parsimony (MP), Maximum Likelihood (ML), and Bayesian Inference (BI) methods) are based on mathematical calculations and IT algorithms [52]. The number of generated phylogenetic trees that should be evaluated to determine the best tree depends substantially on the number of analyzed organisms. The number of possible rooted trees for 32 taxa (i.e., the number of organisms examined in this work) is approximately 1.782 × 10^42^. Because it is impossible to evaluate such a large number of trees, the result in the form of the resulting tree is presented, in the case of the larger number of taxa, on the basis of a very small part of the examined trees (relative to the total number of possible trees). This can be one of the reasons for often not satisfying the reliability of the internal nodes (reliabilities in percents of the internal nodes are presented as numbers close to these nodes in Figure 4). In this work, it is presented that artificial neural networks allow for establishing evolutionary similarities based on recognizing genetic images. This approach (i.e., using artificial neural networks) seems “light”, contrary to the “brutal power of calculation” that is implemented in the NJ, MP, ML, and BI methods [52]. Moreover, because the method proposed in this article is dedicated to the direct analysis (i.e., without calculating hypothetical taxonomic units) of the evolutionary similarity of organisms represented by the leaves of phylogenetic trees, this method can well support and facilitate analyses using phylogenetic trees, for example, by indicating the hypothetical direction of evolution.

### 2.5. Other Analyses

Additionally, analyses of rRNA sequences were performed for organisms previously analyzed based on the amino-acid sequences of cytochrome b and COI. The analyses were performed for organisms whose rRNA sequences were available in the SILVA database [91]. Organism genomes (including rRNA nucleotide sequences) are subject to mutational changes during evolution, and for this reason, rRNA nucleotide sequences vary between organisms, from bacteria to Human (*Homo sapiens*) (http://www.uz.zgora.pl/~akaspers/ijms2026/rRNA.txt (accessed on 23 April 2026)). The rRNA secondary structures of organisms were modeled using the ViennaRNA Web Services [92]. Appendix A.2 presents the studied organisms in the order of their appearance in the virtual line of inheritance of genetic images, starting with Human (*Homo sapiens*) and ending with bacteria. Considering the rRNA secondary structure of these organisms, a “main core” (i.e., a visually recognizable central part of the rRNA secondary structure) is visible (see Appendix A.2). Bacteria have a relatively simple structure, without visible longer lateral elements of the main core. Plants (Date palm (*Phoenix dactylifera*), Grass (*Tripsacum dactyloides*)) also have a relatively simple structure. Analysis of the rRNA secondary structure of the other organisms (Appendix A.2) shows that, during emerging new species, longer lateral elements of the main core can be observed, varying in length and complexity. Of particular interest is the rRNA secondary structure of African bush elephant (*Loxodonta africana*), which has two long lateral elements. Similarities in the shape of the lateral elements are also evident across the organisms, with particularly strong similarities in the secondary structures of Bactrian camel (*Camelus bactrianus*), Horse (*Equus caballus*), and Domestic sheep (*Ovis aries*). Some patterns seen in the rRNA secondary structure are also evident in the secondary structure of organisms belonging to species that emerged in successive stages of evolution. Among the examined organisms (Appendix A.2), the Human (*Homo sapiens*) rRNA secondary structure has the largest number of complex lateral elements. Considering the helices and loops and the visibility of the main core, it can be concluded that the rRNA secondary structures follow a similar overall pattern. Therefore, it can be concluded that changes in genotype do not lead to radical changes in structural phenotype and that these changes occur gradually as new species emerge. The analysis indicates that despite changes in the genotype (i.e., in the rRNA nucleotide sequence), the phenotype (rRNA secondary structure) remains largely conserved throughout evolution, with the complexity of this structure varying over time (i.e., longer and more complex lateral elements of the main core can be observed). In light of the information presented in the Introduction, the appearance of longer lateral elements of the main core and the subsequent increase in their complexity can be interpreted, for example, as a manifestation of the emergence of new functionalities leading to very significant evolutionary changes in the organism. Assuming that the number of lateral elements of the main core, their length, and their degree of complexity indicate evolutionary differences between species, the presented analysis may provide additional confirmation of the reconstructed virtual line of inheritance of genetic images.

## 3. Materials and Methods

Cytochrome b (apocytochrome b) and COI amino-acid sequences selected for this study were taken from NCBI and are available at: http://www.uz.zgora.pl/~akaspers/ijms2026/seq.txt (accessed on 1 April 2026).

rRNA sequences were taken from the SILVA database [91] and are available at: http://www.uz.zgora.pl/~akaspers/ijms2026/rRNA.txt (accessed on 23 April 2026). The rRNA secondary structures were predicted using the ViennaRNA Web Services [92].

Genetic images were recognized using the EvolutionXXI program, which contains an implemented neural network (additionally, see the *Code availability* section). Semihomologous analyses were conducted using the dotPicker program with an implemented semihomologous approach. Phylogenetic trees were generated using the MEGA11 program [93].

### 3.1. A Method of Converting Information Encoded in Amino-Acid Sequences into Genetic Images

Genetic images for each organism were created using the amino-acid sequences of cytochrome b and COI, with each amino-acid assigned a distinct color. As digital representations require binary encoding, each color was mapped to a five-bit binary code (i.e., the color corresponding to alanine (“A”) was encoded as “00001”, aspartate or asparagine (“B”) as “00010”, cysteine (“C”) as “00011”, aspartic acid (“D”) as “00100”, and so on [52]). The cytochrome b and COI amino-acid sequences were aligned to the length of Human (*Homo sapiens*) cytochrome b and COI amino-acid sequences, i.e., to 380 and 513 amino-acids, respectively. The alignment was performed by adding the “-” characters (encoded as “00000”) at the end of shorter sequences or by truncating longer sequences accordingly.

### 3.2. A Method of Recognizing Genetic Images

Artificial neural networks have been used to recognize genetic images. The neural networks have been implemented as full-synapse three-layer (i.e., the input, hidden, and output) neural networks with sigmoid transfer functions [94]. The neural networks were taught using amino-acid sequences of cytochrome b and COI of 32 organisms belonging to different species (the organisms are listed in alphabetical order according to their Latin names): Blue whale (*Balaenoptera musculus*), Bactrian camel (*Camelus bactrianus*), Gray wolf (*Canis lupus*), Indo-Pacific crocodile (*Crocodylus porosus*), Wasp (*Diadegma semiclausum*), Fly (*Drosophila melanogaster*), Horse (*Equus caballus*), Sponge (*Eunapius subterraneus*), Gorilla (*Gorilla gorilla gorilla*), Human (*Homo sapiens*), Chinese hare (*Lepus sinensis*), African bush elephant (*Loxodonta africana*), Atif’s Lycian salamander (*Lyciasalamandra atifi*), Chinese scorpion (*Mesobuthus martensii*), Golden hamster (*Mesocricetus auratus*), Burnett salmon (*Neoceratodus forsteri*), Bacterium 1 (*Nitrobacter vulgaris*), Common octopus (*Octopus vulgaris*), Domestic sheep (*Ovis aries*), Chimpanzee (*Pan troglodytes*), Mushroom (*Phallus echinovolvatus*), Date palm (*Phoenix dactylifera*), Bacterium 2 (*Pseudomonas flexibilis*), Asian rat (*Rattus tanezumi*), Whale shark (*Rhincodon typus*), Maroon-fronted parrot (*Rhynchopsitta terrisi*), Bacterium 3 (*Shewanella xiamenensis*), Atlantic awning clam (*Solemya velum*), Four-horned antelope (*Tetracerus quadricornis*), Pellucid four-tooth moss (*Tetraphis pellucida*), Grass (*Tripsacum dactyloides*), Brown bear (*Ursus arctos*). These organisms, along with the first lines of the FASTA format (containing descriptions of sequences downloaded from NCBI), are listed in Table A1 in Appendix A.1. The downloaded sequences in the FASTA format are also presented at the link: http://www.uz.zgora.pl/~akaspers/ijms2026/seq.txt (accessed on 1 April 2026).

The numbers of neurons in the input layers were equal to: 4465 (i.e., 5 × (380 + 513)) in the neural networks taught using concatenated cytochrome b and COI amino-acid sequences; 1900 (i.e., 5 × 380) in the neural networks taught using cytochrome b amino-acid sequences; and 2565 (i.e., 5 × 513) in the neural network taught using COI amino-acid sequences. The number of outputs was equal to the number of organisms used to teach the neural networks, where each output was associated with one used to teach the organism. That means that each output corresponded to one used to teach the organism. The number of neurons in the hidden layer was calculated by the geometric pyramid rule [95]. According to this rule, for a three-layer network with n inputs and k outputs, the number of neurons in the hidden layer is equal to sqrt(n × k). During the teaching process, binary forms of amino-acid sequences of organisms were put sequentially at the input. The teaching process was repeated until value 1 was obtained at the network output that was associated with the organism that amino-acid sequence was put at the network input and zeros at the other network outputs with a teaching error (SMTP, i.e., root mean squared error) equal to 0.01. As it was checked, SMTP equal to 0.01 gave good generalization ability of the neural networks during recognition. The online backpropagation algorithm was used to teach the neural networks [96]. Two parameters of the teaching, i.e., learning rate and momentum, were set to 0.3 and 0.1, respectively, during teaching neural networks using cytochrome b amino-acid sequences and using COI amino-acid sequences. During ANN teaching using concatenated cytochrome b and COI amino-acid sequences, both learning rate and momentum were set to 0.07. The teaching processes were carried out on the IBM HS23 (2 CPU Xenon E5-2650 × 4 Core, 2.00 GHz, RAM = 12 GB, HDD = 100 GB) computer.

The values of recognition of genetic images (that are within the range [0, 1]) at each neural network output determine the similarities between the genetic images of the organism being recognized and the genetic images of the organisms that were used to teach the neural networks, where 1 indicates maximal and 0 indicates minimal similarity of genetic images.

### 3.3. Semihomologous Approach

In accordance with the semihomologous approach, the occurrence of one-point mutations in the codons of compared amino-acids is the most frequent phenomenon that occurs in homologous proteins [97,98,99]. The semihomologous approach allows for improving (compared to the standard homologous approach) the accuracy of comparing amino-acid sequences. The semihomologous approach assumes the existence of four types of positions [100,101]. The first position type is homologous position (denoted by “R”), which means that the same amino-acids occur at a given position. The next types of positions, i.e., “#”, “$” and “-” indicate that different amino-acids occur at a given position (i.e., in the case of studying evolutionary relationships, at this position amino-acid replacement occurred). Semihomologous positions are denoted by “#” and “$”. “#” means that at a given position there is a one-point mutation of the transition type in the codons of the compared amino-acids. “$” means that at a given position there is a one-point mutation of the transversion type in the codons of the compared amino-acids. “-” means that at a given position there are two or three point mutations in the codons of the compared amino-acids [48,50].

### 3.4. Phylogenetic Tree Generation

Phylogenetic trees were generated using the MEGA11 program [93]. The evolutionary history was inferred by using the Maximum Likelihood method and Jones-Taylor-Thornton (JTT) matrix-based model, along with the activated bootstrap method to determine the reliability of the nodes of the generated trees [89,90,102]. The bootstrap method, with a number of bootstrap replications equal to 500, was used to test phylogeny.

## 4. Conclusions

According to the layered model of the evolution of cellular functionalities, cellular functionalities are located in layers, with new, more evolved cellular functionalities being added to the outer layers [2]. In this way, during the evolution, a layered structure of functionalities is created, in which new functionalities can use, control, and extend already existing functionalities [2]. The results presented in this work provide support for this model by demonstrating that the genomes of organisms belonging to more recently evolved species retain detectable traces of earlier evolutionary stages. To reveal these traces, a novel method based on the recognition of genetic images formed during evolution and derived from amino-acid sequences was introduced. It was demonstrated that artificial neural networks can effectively recognize such genetic images. The analyses show that genetic images of the genetic representatives (i.e., the genetic images derived from cytochrome b and COI amino-acid sequences) of organisms of species from earlier evolutionary stages can be recognized within the genetic images of organisms belonging to more recently evolved species, indicating the inheritance and gradual modification of genetic images. Furthermore, this inheritance enables the determination of the virtual line of inheritance of genetic images, representing a continuum along which inherited genetic images remain detectable. This work can be considered a kind of confirmation that in the genome of organisms is remembered the evolutionary history, manifested through similarities in genetic images, which is consistent with the existence of a specific type of phylogenetic memory that is represented in the cell by the genome and allows for historical functionalities to be stored in the genome [2,103,104]. This memory can re-emerge during cancer transformation and progression, when activities of atavistic functionalities become uncontrolled [2,104,105,106]. In this context, the findings presented in this work may contribute to a better understanding of the mechanisms underlying cancer transformation and progression and may also support, among others, the universal model of cancer transformation and development [2,44,61].

Other key conclusions from this work include:

(a) The concatenation of cytochrome b and COI amino-acid sequences enables artificial neural networks to maintain recognition of genetic images over a greater number of processing steps (additionally, see Remark 4). This indicates that combining these sequences enhances the sensitivity of genetic image recognition by artificial neural networks.
(b) The inheritance of genetic images is observable along the identified virtual line of inheritance of genetic images. This inheritance of genetic images is visible from bacterium to Human (*Homo sapiens*), i.e., from a unicellular, structurally simple organism to a very complex multicellular organism belonging to a species that emerged relatively recently during Earth’s evolutionary history (see Remark 5).
(c) The identification of the virtual line of inheritance of genetic images is challenging, as reflected by the small evolutionary similarities recognized in many cases by ANNs (see, for example, results presented in Table 2, Table 3 and Table 4). This means that during evolution, not only are new functionalities formed, but also existing functionalities are modified, often to a significant extent, which may make it difficult to recognize the inheritance of genetic images.
(d) Three characteristic threshold organisms (i.e., Asian rat, Burnett salmon, and Common octopus) can be distinguished in the reconstructed virtual line of inheritance of genetic images (see Section 2.3).
(e) Bacteria are characterized by high genetic variability, which is why their genetic images are not clearly visible and are difficult to recognize (see results presented in Table 7 and Table 8).
(f) A semihomologous approach confirms the results, i.e., considering a semihomologous approach, organisms belonging to evolutionarily very distant species are characterized by a greater number of transversions (“$”) compared to the number of transitions (“#”). As the evolutionary distance between organisms decreases, the number of homologous (“R”) positions increases, and the difference between the number of transversions (“$”) and the number of transitions (“#”) decreases. As the evolutionary distance continues to decrease, the number of transitions (“#”) reaches the number of transversions (“$”) and then begins to exceed the number of transversions (“$”) (see Section 2.3).
(g) The proposed method allows for determining and studying the evolutionary similarities between organisms without calculating hypothetical taxonomic units, which constitutes a completely new approach in relation to analyses using phylogenetic trees. In this light, the proposed method can be used to support and facilitate the interpretation of phylogenetic trees (see Section 2.4).
(h) To better demonstrate the correctness and effectiveness of the proposed method of recognizing genetic images formed during evolution, other sets of organisms should be tested in the future as a continuation of the current work. It will also be valuable to increase the size of genetic images (by increasing, step-by-step, the number of genes used to generate genetic images) in order to increase the sensitivity of genetic image recognition by artificial neural networks, which should show the real potential of the method proposed in the article.

## Figures and Tables

**Table 1:** (Step 1A of the process) Results of the recognition of the Human (*Homo sapiens*) genetic images after the removal of Human (*Homo sapiens*) from the teaching set of organisms.

Organisms Whose Genetic Images Were Recognized	Cytochrome b and COI ANN & [R/#/$/-]	Cytochrome b ANN & [R/#/$/-]	COI ANN & [R/#/$/-]
Chimpanzee (*Pan troglodytes*)	0.4833 & [862/24/3/4]	0.3865 & [355/20/2/3]	0.8655 & [507/4/1/1]
Gorilla (*Gorilla gorilla gorilla*)	0.1412 & [854/26/8/5]	0.1201 & [350/19/7/4]	0.0875 & [504/7/1/1]

**Table 2:** (Step 2A of the process) Results of the recognition of the Human (*Homo sapiens*) genetic images after additional removal of organisms whose genetic images were recognized in the previous step (i.e., Chimpanzee (*Pan troglodytes*) and Gorilla (*Gorilla gorilla gorilla*)) from the teaching set of organisms.

Organisms Whose Genetic Images Were Recognized	Cytochrome b and COI ANN & [R/#/$/-]	Cytochrome b ANN & [R/#/$/-]	COI ANN & [R/#/$/-]
African bush elephant (*Loxodonta africana*)	0.0148 & [744/59/60/30]	0.0114 & [285/30/42/23]	unrecognized & [459/29/18/7]

**Table 3:** (Step 3A of the process) Results of recognition of the Human (*Homo sapiens*) genetic images after additional removal of the organism whose genetic image was recognized in the previous step (i.e., African bush elephant (*Loxodonta africana*)) from the teaching set of organisms.

Organisms Whose Genetic Images Were Recognized	Cytochrome b and COI ANN & [R/#/$/-]	Cytochrome b ANN & [R/#/$/-]
Gray wolf (*Canis lupus*)	0.0109 & [785/43/43/22]	unrecognized & [314/25/28/13]

**Table 4:** (Step 4A of the process) Results of the recognition of the Human (*Homo sapiens*) genetic images after additional removal of the organism whose genetic image was recognized by the main recognition system (see Remark 4) in the previous step (i.e., Gray wolf (*Canis lupus*)) from the teaching set of organisms.

Organisms Whose Genetic Images Were Recognized	Cytochrome b and COI ANN & [R/#/$/-]
Chinese hare (*Lepus sinensis*)	0.0109 & [771/50/50/22]

**Table 5:** (Step 5A of the process) Results of the recognition of the Chimpanzee (*Pan troglodytes*) genetic images after additional removal of Chinese hare (*Lepus sinensis*) from the teaching set of organisms. Asian rat is marked by “*” as the first organism for which a foreground genetic image was recognized after the change of the reference organism from Human to Chimpanzee.

Organisms Whose Genetic Images Were Recognized	Cytochrome b and COI ANN & [R/#/$/-]
Asian rat (*Rattus tanezumi*) *	0.0174 & [761/53/56/23]
Brown bear (*Ursus arctos*)	0.0108 & [773/53/45/22]

**Table 6:** (Step 9A of the process) Results of the recognition of the Chimpanzee (*Pan troglodytes*) genetic images after additional removal of organisms whose genetic images were recognized by the main recognition system in the previous step (i.e., Maroon-fronted parrot (*Rhynchopsitta terrisi*), Atif’s Lycian salamander (*Lyciasalamandra atifi*), and Indo-Pacific crocodile (*Crocodylus porosus*)) from the teaching set of organisms. Burnett salmon as the threshold organism is marked by “**“.

Organisms Whose Genetic Images Were Recognized	Cytochrome b and COI ANN & [R/#/$/-]
Burnett salmon (*Neoceratodus forsteri*) **	0.0885 & [465/81/144/203]
Whale shark (*Rhincodon typus*)	0.0781 & [469/80/148/196]

**Table 7:** (Step 1B of the process) Results of the recognition of the Bacterium 3 (*Shewanella xiamenensis*) genetic images after the removal of Bacterium 3 (*Shewanella xiamenensis*) from the teaching set of organisms.

Organisms Whose Genetic Images Were Recognized	Cytochrome b and COI ANN & [R/#/$/-]
Bacterium 1 (*Nitrobacter vulgaris*)	0.0084 & [44/114/172/563]

**Table 8:** (Step 2B of the process) Results of the recognition of the Bacterium 3 (*Shewanella xiamenensis*) genetic images after additional removal of Bacterium 1 (*Nitrobacter vulgaris*) from the teaching set of organisms.

Organisms Whose Genetic Images Were Recognized	Cytochrome b and COI ANN & [R/#/$/-]
Bacterium 2 (*Pseudomonas flexibilis*)	0.0062 & [67/116/246/464]

**Table 9:** (Step 3B of the process) Results of the recognition of the Bacterium 3 (*Shewanella xiamenensis*) genetic images after additional removal of Bacterium 2 (*Pseudomonas flexibilis*) from the teaching set of organisms.

Organisms Whose Genetic Images Were Recognized	Cytochrome b and COI ANN & [R/#/$/-]
Grass (*Tripsacum dactyloides*)	0.0100 & [55/133/264/441]

**Table 10:** (Step 7B of the process) Results of the recognition of the Bacterium 3 (*Shewanella xiamenensis*) genetic images after additional removal of organisms whose genetic images were recognized by the main recognition system in the previous step (i.e., Chinese scorpion (*Mesobuthus martensii*), Fly (*Drosophila melanogaster*), Mushroom (*Phallus echinovolvatus*), and Wasp (*Diadegma semiclausum*)) from the teaching set of organisms. Common octopus as the threshold organism is marked by “***“.

Organisms Whose Genetic Images Were Recognized	Cytochrome b and COI ANN & [R/#/$/-]
Common octopus (*Octopus vulgaris*) ***	0.1884 & [71/133/245/444]

## Data Availability

Cytochrome b (apocytochrome b) and COI amino-acid sequences selected for this study were taken from NCBI and are available at: http://www.uz.zgora.pl/~akaspers/ijms2026/seq.txt (accessed on 1 April 2026). rRNA sequences were taken from the SILVA database [91] and are available at: http://www.uz.zgora.pl/~akaspers/ijms2026/rRNA.txt (accessed on 23 April 2026). Code availability—The EvolutionXXI and dotPicker programs (available at http://www.uz.zgora.pl/~akaspers/ijms2026/pgms.zip (accessed on 1 April 2026)) have been written by the author of this article. The EvolutionXXI program has been written in Java using the Joone framework, and it can be run on any platform with an installed Java Virtual Machine (JVM). The dotPicker program has been written in C#, and it can be run on Windows with the installed .NET Framework.

## Appendix A

### Appendix A.1

**Table A1:** 32 organisms whose cytochrome b/apocytochrome b and COI amino-acid sequences were used to teach the neural networks. The organisms are listed in alphabetical order according to their Latin names.

No.	Organisms and Descriptions of Sequences (i.e., the First Lines of Downloaded Sequences in the FASTA Format)
1	Blue whale (*Balaenoptera musculus*) >gi|745749243|gb|AJD22663.1| cytochrome b (mitochondrion) [Balaenoptera musculus] >NP_007058.1 cytochrome c oxidase subunit I (mitochondrion) [Balaenoptera musculus]
2	Bactrian camel (*Camelus bactrianus*) >gi|150375660|ref|YP_001315071.1| cytochrome b (mitochondrion) [Camelus bactrianus] >ABO70689.1 cytochrome c oxidase subunit I (mitochondrion) [Camelus bactrianus]
3	Gray wolf (*Canis lupus*) >gi|575524860|gb|AHG98585.1| cytochrome b (mitochondrion) [Canis lupus] >BAJ05711.1 cytochrome c oxidase subunit I (mitochondrion) [Canis lupus]
4	Indo-Pacific crocodile (*Crocodylus porosus*) >gi|81295124|emb|CAH18616.1| cytochrome b (mitochondrion) [Crocodylus porosus] >ABB72515.1 cytochrome c oxidase subunit I (mitochondrion) [Crocodylus porosus]
5	Wasp (*Diadegma semiclausum*) >gi|237869082|ref|YP_002907411.1| cytochrome b (mitochondrion) [Diadegma semiclausum] >ACF35057.1 cytochrome c oxidase subunit I (mitochondrion) [Diadegma semiclausum]
6	Fly (*Drosophila melanogaster*) >AAC47822.1 cytochrome b (mitochondrion) [Drosophila melanogaster] >AAF77227.1 cytochrome c oxidase subunit I (mitochondrion) [Drosophila melanogaster]
7	Horse (*Equus caballus*) >gi|1001185184|gb|AML80388.1| cytochrome b (mitochondrion) [Equus caballus] >ACH95421.1 cytochrome c oxidase subunit I (mitochondrion) [Equus caballus]
8	Sponge (*Eunapius subterraneus*) >gi|359422215|ref|YP_004935622.1| cytochrome b (mitochondrion) [Eunapius subterraneus] >YP_004935628.1 cytochrome c oxidase subunit I (mitochondrion) [Eunapius subterraneus]
9	Gorilla (*Gorilla gorilla gorilla*) >YP_002120670.1 cytochrome b (mitochondrion) [Gorilla gorilla gorilla] >YP_002120661.1 cytochrome c oxidase subunit I (mitochondrion) [Gorilla gorilla gorilla]
10	Human (*Homo sapiens*) >gi|698352801|gb|AIT96887.1| cytochrome b (mitochondrion) [Homo sapiens] >BAA07292.1 cytochrome c oxidase subunit 1 (mitochondrion) [Homo sapiens]
11	Chinese hare (*Lepus sinensis*) >gi|6692082|emb|CAB65749.1| cytochrome b (mitochondrion) [Lepus sinensis] >YP_009093563.1 cytochrome c oxidase subunit I (mitochondrion) [Lepus sinensis]
12	African bush elephant (*Loxodonta africana*) >gi|59859518|gb|AAX09500.1| cytochrome b (mitochondrion) [Loxodonta africana] >NP_009281.1 cytochrome c oxidase subunit I (mitochondrion) [Loxodonta africana]
13	Atif’s Lycian salamander (*Lyciasalamandra atifi*) >AAK43358.1 cytochrome b (mitochondrion) [Lyciasalamandra atifi] >AAK43348.1 cytochrome c oxidase subunit I (mitochondrion) [Lyciasalamandra atifi]
14	Chinese scorpion (*Mesobuthus martensii*) >gi|155969672|ref|YP_001427353.1| cytochrome b (mitochondrion) [Mesobuthus martensii] >ABC71907.1 cytochrome c oxidase subunit I (mitochondrion) [Mesobuthus martensii]
15	Golden hamster (*Mesocricetus auratus*) >gi|260150998|ref|YP_003208313.1| cytochrome b (mitochondrion) [Mesocricetus auratus] >ACC86262.1 cytochrome c oxidase subunit 1 (mitochondrion) [Mesocricetus auratus]
16	Burnett salmon (*Neoceratodus forsteri*) >AAL08601.1 cytochrome b (mitochondrion) [Neoceratodus forsteri] >NP_387474.1 cytochrome c oxidase subunit I (mitochondrion) [Neoceratodus forsteri]
17	Bacterium 1 (*Nitrobacter vulgaris*) >OPH81403.1 cytochrome B [Nitrobacter vulgaris] >OPH81312.1 cytochrome c oxidase subunit I [Nitrobacter vulgaris]
18	Common octopus (*Octopus vulgaris*) >gi|53793898|ref|YP_112443.1| cytochrome b (mitochondrion) [Octopus vulgaris] >YP_112436.1 cytochrome c oxidase subunit I (mitochondrion) [Octopus vulgaris]
19	Domestic sheep (*Ovis aries*) >gi|873817793|gb|AKP54616.1| cytochrome b (mitochondrion) [Ovis aries] >AKT25940.1 cytochrome c oxidase subunit I (mitochondrion) [Ovis aries]
20	Chimpanzee (*Pan troglodytes*) >NP_008198.1 cytochrome b (mitochondrion) [Pan troglodytes] >NP_008188.1 cytochrome c oxidase subunit I (mitochondrion) [Pan troglodytes]
21	Mushroom (*Phallus echinovolvatus*) >QLD96661.1 apocytochrome b (mitochondrion) [Phallus echinovolvatus] >YP_009912203.1 cytochrome c oxidase subunit 1 (mitochondrion) [Phallus echinovolvatus]
22	Date palm (*Phoenix dactylifera*) >gi|372450209|ref|YP_005090362.1| apocytochrome b (mitochondrion) [Phoenix dactylifera] >YP_005090365.1 cytochrome c oxidase subunit 1 (mitochondrion) [Phoenix dactylifera]
23	Bacterium 2 (*Pseudomonas flexibilis*) >KHO65136.1 cytochrome B [Pseudomonas flexibilis] >KHL68328.1 cytochrome c oxidase subunit I [Pseudomonas flexibilis]
24	Asian rat (*Rattus tanezumi*) >gi|371754534|gb|AEX55475.1| cytochrome b (mitochondrion) [Rattus tanezumi] >ABX45300.1 cytochrome c oxidase subunit 1 (mitochondrion) [Rattus tanezumi]
25	Whale shark (*Rhincodon typus*) >gi|584593628|ref|YP_009002145.1| cytochrome b (mitochondrion) [Rhincodon typus] >AVI15574.1 cytochrome c oxidase subunit I (mitochondrion) [Rhincodon typus]
26	Maroon-fronted parrot (*Rhynchopsitta terrisi*) >gi|525341196|ref|YP_008239403.1| cytochrome b (mitochondrion) [Rhynchopsitta terrisi] >YP_008239394.1 cytochrome c oxidase subunit I (mitochondrion) [Rhynchopsitta terrisi]
27	Bacterium 3 (*Shewanella xiamenensis*) >BDA62316.1 cytochrome b [Shewanella xiamenensis] >UML93620.1 cytochrome c oxidase subunit I [Shewanella xiamenensis]
28	Atlantic awning clam (*Solemya velum*) >gi|383931376|ref|YP_006073353.1| cytochrome b (mitochondrion) [Solemya velum] >AFG18166.1 cytochrome c oxidase subunit 1 (mitochondrion) [Solemya velum]
29	Four-horned antelope (*Tetracerus quadricornis*) >gi|5777910|gb|AAD51425.1|AF036274_1 cytochrome b (mitochondrion) [Tetracerus quadricornis] >YP_007627201.1 cytochrome c oxidase subunit I (mitochondrion) [Tetracerus quadricornis]
30	Pellucid four-tooth moss (*Tetraphis pellucida*) >AHI16091.1 apocytochrome b (mitochondrion) [Tetraphis pellucida] >YP_009040960.1 cytochrome c oxidase subunit 1 (mitochondrion) [Tetraphis pellucida]
31	Grass (*Tripsacum dactyloides*) >ABI74645.1 apocytochrome b (mitochondrion) [Tripsacum dactyloides] >YP_762510.1 cytochrome c oxidase subunit 1 (mitochondrion) [Tripsacum dactyloides]
32	Brown bear (*Ursus arctos*) >gi|556502668|emb|CDF66369.1| cytochrome b (mitochondrion) [Ursus arctos] >BAN57868.1 cytochrome c oxidase subunit 1 (mitochondrion) [Ursus arctos]

**Table A2:** (Step 6A of the process) Results of the recognition of the Chimpanzee (*Pan troglodytes*) genetic images after additional removal of organisms whose genetic images were recognized by the main recognition system (i.e., a system consisting of the ANNs taught using concatenated cytochrome b and COI amino-acid sequences) in the previous step (i.e., Asian rat (*Rattus tanezumi*) and Brown bear (*Ursus arctos*)) from the teaching set of organisms.

Organisms Whose Genetic Images Were Recognized	Cytochrome b and COI ANN & [R/#/$/-]
Golden hamster (*Mesocricetus auratus*)	0.0420 & [751/50/62/30]
Bactrian camel (*Camelus bactrianus*)	0.0419 & [766/51/50/26]
Horse (*Equus caballus*)	0.0237 & [773/50/46/24]

**Table A3:** (Step 7A of the process) Results of the recognition of the Chimpanzee (*Pan troglodytes*) genetic images after additional removal of organisms whose genetic images were recognized by the main recognition system in the previous step (i.e., Golden hamster (*Mesocricetus auratus*), Bactrian camel (*Camelus bactrianus*), and Horse (*Equus caballus*)) from the teaching set of organisms.

Organisms Whose Genetic Images Were Recognized	Cytochrome b and COI ANN & [R/#/$/-]
Four-horned antelope (*Tetracerus quadricornis*)	0.1149 & [763/49/57/24]
Domestic sheep (*Ovis aries*)	0.0727 & [760/48/59/26]
Blue whale (*Balaenoptera musculus*)	0.0705 & [758/51/54/30]

**Table A4:** (Step 8A of the process) Results of the recognition of the Chimpanzee (*Pan troglodytes*) genetic images after additional removal of organisms whose genetic images were recognized by the main recognition system in the previous step (i.e., Four-horned antelope (*Tetracerus quadricornis*), Domestic sheep (*Ovis aries*), and Blue whale (*Balaenoptera musculus*)) from the teaching set of organisms.

Organisms Whose Genetic Images Were Recognized	Cytochrome b and COI ANN & [R/#/$/-]
Maroon-fronted parrot (*Rhynchopsitta terrisi*)	0.0415 & [481/84/135/193]
Atif’s Lycian salamander (*Lyciasalamandra atifi*)	0.0163 & [481/85/140/187]
Indo-Pacific crocodile (*Crocodylus porosus*)	0.0103 & [456/94/148/195]

**Table A5:** (Step 10A of the process) Results of the recognition of the Chimpanzee (*Pan troglodytes*) genetic images after additional removal of organisms whose genetic images were recognized by the main recognition system in the previous step (i.e., Burnett salmon (*Neoceratodus forsteri*) and Whale shark (*Rhincodon typus*)) from the teaching set of organisms.

Organisms Whose Genetic Images Were Recognized	Cytochrome b and COI ANN & [R/#/$/-]
Mushroom (*Phallus echinovolvatus*)	0.0612 & [208/114/227/344]

**Table A6:** (Step 11A of the process) Results of the recognition of the Chimpanzee (*Pan troglodytes*) genetic images after additional removal of the organism whose genetic image was recognized by the main recognition system in the previous step (i.e., Mushroom (*Phallus echinovolvatus*)) from the teaching set of organisms.

Organisms Whose Genetic Images Were Recognized	Cytochrome b and COI ANN & [R/#/$/-]
Bacterium 3 (*Shewanella xiamenensis*)	0.0150 & [67/136/228/462]
Bacterium 2 (*Pseudomonas flexibilis*)	0.0136 & [67/116/246/464]
Pellucid four-tooth moss (*Tetraphis pellucida*)	0.0119 & [63/131/264/435]

**Table A7:** (Step 4B of the process) Results of the recognition of the Bacterium 3 (*Shewanella xiamenensis*) genetic images after additional removal of the organism whose genetic image was recognized by the main recognition system in the previous step (i.e., Grass (*Tripsacum dactyloides*)) from the teaching set of organisms.

Organisms Whose Genetic Images Were Recognized	Cytochrome b and COI ANN & [R/#/$/-]
Date palm (*Phoenix dactylifera*)	0.0191 & [57/140/256/440]

**Table A8:** (Step 5B of the process) Results of the recognition of the Bacterium 3 (*Shewanella xiamenensis*) genetic images after additional removal of the organism whose genetic image was recognized by the main recognition system in the previous step (i.e., Date palm (*Phoenix dactylifera*)) from the teaching set of organisms.

Organisms Whose Genetic Images Were Recognized	Cytochrome b and COI ANN & [R/#/$/-]
Pellucid four-tooth moss (*Tetraphis pellucida*)	0.0171 & [63/131/264/435]
Atlantic awning clam (*Solemya velum*)	0.0158 & [68/132/251/442]
Sponge (*Eunapius subterraneus*)	0.0121 & [64/141/229/459]

**Table A9:** (Step 6B of the process) Results of the recognition of the Bacterium 3 (*Shewanella xiamenensis*) genetic images after additional removal of organisms whose genetic images were recognized by the main recognition system in the previous step (i.e., Pellucid four-tooth moss (*Tetraphis pellucida*), Atlantic awning clam (*Solemya velum*), and Sponge (*Eunapius subterraneus*)) from the teaching set of organisms.

Organisms Whose Genetic Images Were Recognized	Cytochrome b and COI ANN & [R/#/$/-]
Chinese scorpion (*Mesobuthus martensii*)	0.0340 & [73/146/254/420]
Fly (*Drosophila melanogaster*)	0.0200 & [74/133/261/425]
Mushroom (*Phallus echinovolvatus*)	0.0138 & [208/114/227/344]
Wasp (*Diadegma semiclausum*)	0.0101 & [71/146/227/449]

**Table A10:** (Step 8B of the process) Results of the recognition of the Bacterium 3 (*Shewanella xiamenensis*) genetic images after additional removal of the organism whose genetic image was recognized by the main recognition system in the previous step (i.e., Common octopus (*Octopus vulgaris*)) from the teaching set of organisms.

Organisms Whose Genetic Images Were Recognized	Cytochrome b and COI ANN & [R/#/$/-]
Indo-Pacific crocodile (*Crocodylus porosus*)	0.0326 & [456/94/148/195]

**Table A11:** (Step 9B of the process) Results of the recognition of the Bacterium 3 (*Shewanella xiamenensis*) genetic images after additional removal of the organism whose genetic image was recognized by the main recognition system in the previous step (i.e., Indo-Pacific crocodile (*Crocodylus porosus*)) from the teaching set of organisms.

Organisms Whose Genetic Images Were Recognized	Cytochrome b and COI ANN & [R/#/$/-]
Whale shark (*Rhincodon typus*)	0.0296 & [469/80/148/196]

**Table A12:** (Step 10B of the process) Results of the recognition of the Bacterium 3 (*Shewanella xiamenensis*) genetic images after additional removal of the organism whose genetic image was recognized by the main recognition system in the previous step (i.e., Whale shark (*Rhincodon typus*)) from the teaching set of organisms.

Organisms Whose Genetic Images Were Recognized	Cytochrome b and COI ANN & [R/#/$/-]
Maroon-fronted parrot (*Rhynchopsitta terrisi*)	0.0361 & [481/84/135/193]
Burnett salmon (*Neoceratodus forsteri*)	0.0247 & [465/81/144/203]
Atif’s Lycian salamander (*Lyciasalamandra atifi*)	0.0143 & [481/85/140/187]

**Table A13:** (Step 11B of the process) Results of the recognition of the Bacterium 3 (*Shewanella xiamenensis*) genetic images after additional removal of organisms whose genetic images were recognized by the main recognition system in the previous step (i.e., Maroon-fronted parrot (*Rhynchopsitta terrisi*), Burnett salmon (*Neoceratodus forsteri*), and Atif’s Lycian salamander (*Lyciasalamandra atifi*)) from the teaching set of organisms.

Organisms Whose Genetic Images Were Recognized	Cytochrome b and COI ANN & [R/#/$/-]
Golden hamster (*Mesocricetus auratus*)	0.0127 & [751/50/62/30]
African bush elephant (*Loxodonta africana*)	0.0102 & [744/59/60/30]

**Table A14:** (Step 12B of the process) Results of the recognition of the Bacterium 3 (*Shewanella xiamenensis*) genetic images after additional removal of organisms whose genetic images were recognized by the main recognition system in the previous step (i.e., Golden hamster (*Mesocricetus auratus*) and African bush elephant (*Loxodonta africana*)) from the teaching set of organisms.

Organisms Whose Genetic Images Were Recognized	Cytochrome b and COI ANN & [R/#/$/-]
Asian rat (*Rattus tanezumi*)	0.0345 & [761/53/56/23]
Gorilla (*Gorilla gorilla gorilla*)	0.0221 & [854/26/8/5]
Chimpanzee (*Pan troglodytes*)	0.0212 & [862/24/3/4]

**Table A15:** (Step 13B of the process) Results of the recognition of the Bacterium 3 (*Shewanella xiamenensis*) genetic images after additional removal of organisms whose genetic images were recognized by the main recognition system in the previous step (i.e., Asian rat (*Rattus tanezumi*), Gorilla (*Gorilla gorilla gorilla*), and Chimpanzee (*Pan troglodytes*)) from the teaching set of organisms.

Organisms Whose Genetic Images Were Recognized	Cytochrome b and COI ANN & [R/#/$/-]
Human (*Homo sapiens*)	0.1509 & [893/0/0/0]
Blue whale (*Balaenoptera musculus*)	0.0180 & [758/51/54/30]
Brown bear (*Ursus arctos*)	0.0104 & [773/53/45/22]

### Appendix A.2

Secondary structures of rRNA (minimum free energy secondary structures) predicted using the ViennaRNA Web Services [92].

** **

Human, *Homo sapiens*

>AADC01166060.3659.5519 Eukaryota; Amorphea; Obazoa; Opisthokonta; Holozoa; Choanozoa; Metazoa; Animalia; BCP clade; Bilateria; Deuterostomia; Chordata; Vertebrata; Gnathostomata; Euteleostomi; Tetrapoda; Mammalia; Homo sapiens (human).

** **

Chimpanzee, *Pan troglodytes*

>AACZ04068423.75350.77214 Eukaryota; Amorphea; Obazoa; Opisthokonta; Holozoa; Choanozoa; Metazoa; Animalia; BCP clade; Bilateria; Deuterostomia; Chordata; Vertebrata; Gnathostomata; Euteleostomi; Tetrapoda; Mammalia; Pan troglodytes (chimpanzee).

** **

Gorilla, *Gorilla gorilla gorilla*

>CABD030100647.887.2742 Eukaryota; Amorphea; Obazoa; Opisthokonta; Holozoa; Choanozoa; Metazoa; Animalia; BCP clade; Bilateria; Deuterostomia; Chordata; Vertebrata; Gnathostomata; Euteleostomi; Tetrapoda; Mammalia; Gorilla gorilla gorilla (western lowland gorilla).

** **

African bush elephant, *Loxodonta africana*

>AAGU03092596.576.2373 Eukaryota; Amorphea; Obazoa; Opisthokonta; Holozoa; Choanozoa; Metazoa; Animalia; BCP clade; Bilateria; Deuterostomia; Chordata; Vertebrata; Gnathostomata; Euteleostomi; Tetrapoda; Mammalia; Loxodonta africana (African savanna elephant).

** **

Golden hamster, *Mesocricetus auratus*

>GEMX01008541.1.1427 Eukaryota; Amorphea; Obazoa; Opisthokonta; Holozoa; Choanozoa; Metazoa; Animalia; BCP clade; Bilateria; Deuterostomia; Chordata; Vertebrata; Gnathostomata; Euteleostomi; Tetrapoda; Mammalia; Mesocricetus auratus (golden hamster).

** **

Bactrian camel, *Camelus bactrianus*

>CAOW010004432.124.1985 Eukaryota; Amorphea; Obazoa; Opisthokonta; Holozoa; Choanozoa; Metazoa; Animalia; BCP clade; Bilateria; Deuterostomia; Chordata; Vertebrata; Gnathostomata; Euteleostomi; Tetrapoda; Mammalia; Camelus bactrianus (Bactrian camel).

** **

Horse, *Equus caballus*

>AAWR02050020.103.1963 Eukaryota; Amorphea; Obazoa; Opisthokonta; Holozoa; Choanozoa; Metazoa; Animalia; BCP clade; Bilateria; Deuterostomia; Chordata; Vertebrata; Gnathostomata; Euteleostomi; Tetrapoda; Mammalia; Equus caballus (horse).

** **

Domestic *sheep, Ovis aries*

>KY129860.1.1869 Eukaryota; Amorphea; Obazoa; Opisthokonta; Holozoa; Choanozoa; Metazoa; Animalia; BCP clade; Bilateria; Deuterostomia; Chordata; Vertebrata; Gnathostomata; Euteleostomi; Tetrapoda; Mammalia; Ovis aries (sheep).

** **

Indo-Pacific crocodile, *Crocodylus porosus*

>JRXG01085526.12838.14647 Eukaryota; Amorphea; Obazoa; Opisthokonta; Holozoa; Choanozoa; Metazoa; Animalia; BCP clade; Bilateria; Deuterostomia; Chordata; Vertebrata; Gnathostomata; Euteleostomi; Tetrapoda; Crocodylia; Crocodylus porosus (Australian saltwater crocodile).

** **

Burnett salmon, *Neoceratodus forsteri*

>KJ784395.1.1738 Eukaryota; Amorphea; Obazoa; Opisthokonta; Holozoa; Choanozoa; Metazoa; Animalia; BCP clade; Bilateria; Deuterostomia; Chordata; Vertebrata; Gnathostomata; Euteleostomi; Sarcopterygii; Dipnoi; Neoceratodus forsteri (Australian lungfish).

** **

Whale shark, *Rhincodon typus*

>LVEK02000095.4102.5906 Eukaryota; Amorphea; Obazoa; Opisthokonta; Holozoa; Choanozoa; Metazoa; Animalia; BCP clade; Bilateria; Deuterostomia; Chordata; Vertebrata; Gnathostomata; Chondrichthyes; Elasmobranchii; Batoidea; Rhincodon typus (whale shark).

** **

Common octopus, *Octopus vulgaris*

>FJ617439.1.422 Eukaryota; Amorphea; Obazoa; Opisthokonta; Holozoa; Choanozoa; Metazoa; Animalia; BCP clade; Bilateria; Protostomia; Lophotrochozoa; Mollusca; Cephalopoda; Coleoidea; Decapodiformes; Teuthida; Octopus vulgaris (common octopus).

** **

Chinese scorpion, *Mesobuthus martensii*

>FJ948787.8246.10059 Eukaryota; Amorphea; Obazoa; Opisthokonta; Holozoa; Choanozoa; Metazoa; Animalia; BCP clade; Bilateria; Protostomia; Ecdysozoa; Arthropoda; Chelicerata; Arachnida; Scorpiones; Mesobuthus martensii (Chinese scorpion).

** **

Fly, *Drosophila melanogaster*

>JAQD01000152.1022.2897 Eukaryota; Amorphea; Obazoa; Opisthokonta; Holozoa; Choanozoa; Metazoa; Animalia; BCP clade; Bilateria; Protostomia; Ecdysozoa; Arthropoda; Hexapoda; Insecta; Pterygota; Neoptera; Diptera; Drosophila melanogaster (fruit fly).

** **

Pellucid four-tooth moss, *Tetraphis pellucida*

>Y17604.1.1813 Eukaryota; Archaeplastida; Chloroplastida; Charophyta; Phragmoplastophyta; Streptophyta; Embryophyta; Bryophyta;Tetraphidales; Tetraphis;Tetraphis pellucida.

** **

Atlantic awning clam, *Solemya velum*

>AF120524.1.1771 Eukaryota; Amorphea; Obazoa; Opisthokonta; Holozoa; Choanozoa; Metazoa; Animalia; BCP clade; Bilateria; Protostomia; Lophotrochozoa; Mollusca; Bivalvia; Protobranchia; Solemyoida; Solemya velum (Atlantic awning clam).

** **

Sponge, *Eunapius subterraneus*

>FJ715438.1.1616 Eukaryota; Amorphea; Obazoa; Opisthokonta; Holozoa; Choanozoa; Metazoa; Porifera; Demospongiae; Spongillida; Eunapius subterraneus (Ogulin cave sponge).

** **

Date palm, *Phoenix dactylifera*

>AY012354.1.1703 Eukaryota; Archaeplastida; Chloroplastida; Charophyta; Phragmoplastophyta; Streptophyta; Embryophyta; Tracheophyta; Spermatophyta; Magnoliophyta; Liliopsida; Arecales; Phoenix; Phoenix dactylifera (date palm).

** **

Grass, *Tripsacum dactyloides*

>DQ984517.668079.670042 Bacteria; Pseudomonadota; Alphaproteobacteria; Rickettsiales; Mitochondria; Incertae Sedis; Tripsacum dactyloides (gama grass).

** **

Bacterium 3, *Shewanella xiamenensis*

>FJ589031.1.1535 Bacteria; Pseudomonadota; Gammaproteobacteria; Enterobacterales; Shewanellaceae; Shewanella; Shewanella xiamenensis.

** **

Bacterium 1, *Nitrobacter vulgaris*

>MWPQ01000010.49802.51298 Bacteria; Pseudomonadota; Alphaproteobacteria; Hyphomicrobiales; Xanthobacteraceae; Nitrobacter; Nitrobacter vulgaris.

** **

Bacterium 2, *Pseudomonas flexibilis*

>KP973996.1.1530 Bacteria; Pseudomonadota; Gammaproteobacteria; Pseudomonadales; Pseudomonadaceae; Pseudomonas; Pseudomonas flexibilis.
